# Herbal Medicine Yukgunja-Tang for Functional Dyspepsia: A Protocol for a Randomized, Controlled, Multicenter Clinical Trial

**DOI:** 10.3390/healthcare11101456

**Published:** 2023-05-17

**Authors:** Boram Lee, Na-Yeon Ha, Hyo-Ju Park, Ae-Ran Kim, O-Jin Kwon, Jung-Hyo Cho, Seon Mi Shin, Jinsung Kim, Changsop Yang

**Affiliations:** 1 KM Science Research Division, Korea Institute of Oriental Medicine, Daejeon 34054, Republic of Korea; 2 Division of Digestive Diseases, Department of Internal Korean Medicine, Kyung Hee University Medical Center, Seoul 02447, Republic of Korea; 3 Clinical Research Coordinating Team, Korea Institute of Oriental Medicine, Daejeon 34054, Republic of Korea; 4 Department of Internal Medicine, Daejeon Korean Medicine Hospital of Daejeon University, Daejeon 35235, Republic of Korea; 5 Department of Internal Medicine, College of Korean Medicine, Semyung University, Jecheon-si 27136, Republic of Korea; 6 Department of Gastroenterology, Kyung Hee University College of Korean Medicine, Kyung Hee University Medical Center, Seoul 02447, Republic of Korea

**Keywords:** functional dyspepsia, Yukgunja-tang, Rikkunshito, Liujunzi-tang, herbal medicine, randomized controlled trial

## Abstract

The herbal medicine Yukgunja-tang has been widely used for the treatment of functional dyspepsia (FD) in the clinical setting of East Asian traditional medicine. This paper presents a protocol for a randomized, assessor-blind, controlled, multicenter, three-arm parallel clinical trial comparing the effectiveness, safety, and cost-effectiveness of Yukgunja-tang with Pyeongwi-san and usual care. A total of 140 participants with Rome IV-diagnosed FD will be randomly assigned to either the Yukgunja-tang (n = 56), Pyeongwi-san (n = 56), or usual care (n = 28) groups. All participants will be educated on dietary guidelines for FD patients. Participants in the Yukgunja-tang and Pyeongwi-san groups will take investigational products for 6 weeks. All participants will be assessed for clinical parameters at weeks 0, 3, 6, 9, and 24. The primary outcome will be measured on the total dyspepsia symptom scale, and the secondary outcome will include the single dyspepsia symptom scale, overall treatment effect, the visual analog scale for dyspepsia, FD-related quality of life, hospital anxiety and depression scale, EuroQol-5 dimension, pattern identification, and serum levels of acyl-ghrelin and deacyl-ghrelin. Adverse events and laboratory tests will be monitored for safety assessment. The results will provide evidence of the effectiveness, safety, and cost-effectiveness of Yukgunja-tang in the treatment of FD.

## 1. Introduction

Functional dyspepsia (FD) is a chronic non-communicable gastrointestinal disease characterized by the following various symptoms: postprandial fullness, early satiety, and epigastric pain or burning without evidence of structural abnormality [1]. The prevalence of FD is 5–11% worldwide [2], and 7.7% in Korea [3]. More than 50% of patients with FD report that it interferes with their daily life, and thus it is a disease of great socioeconomic burden [4]. In Western medicine, various drugs are used to relieve the symptoms of indigestion. However, there are limitations to conventional treatment for treating various symptoms of FD, and the demand for complementary and integrative medicine is increasing globally [5].

Herbal medicine, one of the complementary and integrative forms of medicine, has been widely used for the treatment of various diseases, including FD, worldwide [6,7]. Because herbal medicines are typical multitarget multicomponent agents [8], they have promising potential for the treatment of FD, which shows various pathological mechanisms and symptoms. According to the statistical data of the Korea Health Insurance Review and Assessment Service, FD ranked 8th among outpatients at Korean medical institutions in 2021, and many people are receiving Korean medicine treatment such as herbal medicine and acupuncture for FD [9].

Yukgunja-tang (YGJ; Rikkunshito in Japanese, Liujunzi-tang in Chinese), one of the representative herbal medicines used to treat FD, is an herbal prescription consisting of eight herbs: Pinelliae Tuber, Citri Unshius Pericarpium, Ginseng Radix, Atractylodis Rhizoma Alba, Poria Sclerotium, Glycyrrhizae Radix et Rhizoma, Zingiberis Rhizoma Recens, and Zizyphi Fructus. In particular, according to a survey conducted on 349 clinical Korean medicine doctors in 2017, YGJ was the most frequently used herbal prescription among noninsured herbal medicines in Korea [10]. However, despite its active use, YGJ is not covered by health insurance in Korea, causing a burden on one’s private medical expenses.

In addition, although several clinical studies of YGJ for the treatment of FD have already been carried out in other East Asian countries, including Japan and China [11,12,13,14], there is no large-scale clinical trial to investigate the effectiveness of YGJ on FD, reflecting the Korean medicine clinical field. According to a recent systematic review, the administration of YGJ, alone or in collaboration with Western medicine, can be an effective and safe treatment option for FD [15]. However, the authors concluded that there was uncertain evidence due to the low quality of included studies [15]. Therefore, a well-designed, high-quality randomized controlled trial (RCT) is needed to verify the effectiveness of YGJ in the treatment of FD.

In this study, we aimed to evaluate the therapeutic effect of YGJ on FD by comparing it with the usual care control group. In addition, we will compare the effectiveness of YGJ with that of Pyeongwi-san (PWS). PWS, an herbal medicine that is commonly used for the treatment of FD, has been shown to be effective in the treatment of FD through previous clinical trials [16,17]. Furthermore, it is the most frequently used herbal medicine in Korea for the treatment of FD within the scope of national health insurance [7], and is therefore an appropriate control group to evaluate the noninferiority and cost-effectiveness of a specific herbal medicine for the treatment of FD. We aimed to analyze the noninferiority and cost-effectiveness of YGJ compared with PWS, exploring the possibilities of YGJ being listed for insurance coverage in Korea. To our knowledge, this study is the first to test the noninferiority and cost-effectiveness of YGJ, which is actively used for FD treatment in clinical settings.

## 2. Methods

### 2.1. Study Design

**Figure 1:**
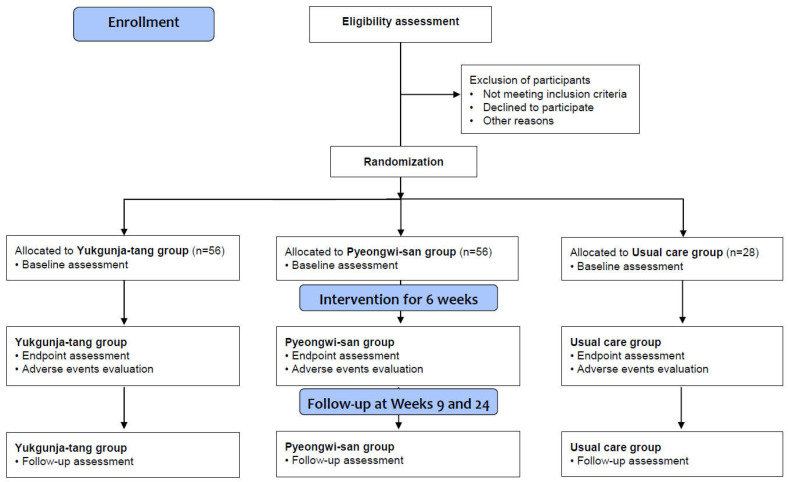
Flow chart of the trial process.

A randomized, assessor-blind, three-arm, parallel-group, multicenter clinical trial will be conducted at the Kyung Hee University Korean Medicine Hospital, Daejeon Korean Medicine Hospital of Daejeon University, and Semyung University Korean Medicine Hospital in the Republic of Korea from June 2022 to December 2023. The trial includes 6 weeks of medication or usual care (week 0 to 6) and a follow-up of 18 weeks (from week 6 to 24), following 2 weeks of a run-in period after the screening visit (Table 1 and Figure 1). It will be performed according to the declaration of Helsinki and good clinical practice guidelines. Investigators will provide detailed trial information to participants through standardized interviews prior to participation, and they will obtain written informed consent from all participants. The participants will be allowed to withdraw from the study at any time without penalties. We report this protocol in accordance with the standard protocol items: the recommendations for interventional trials 2013 statement [18].

### 2.2. Participant Recruitment

We will recruit participants through offline and online recruitment advertisements, including posting posters on bulletin boards and websites of trial institutions. If participant registration is delayed, local advertisements in local public transportation and newspapers will be placed.

### 2.3. Inclusion Criteria

- Adults aged 19–75 years at the time of screening visit;
- Diagnosed with FD according to Rome IV criteria (having one or more symptoms of postprandial fullness, early satiety, epigastric pain, and epigastric burning without organic findings, occurred at least 6 months ago and persisted for at least past 3 months);
- The intensity of dyspeptic symptoms measured by a 0–100 mm visual analog scale (VAS) is at least 40 points; and
- Voluntarily signed written informed consent approved by the institutional review board (IRB).

### 2.4. Exclusion Criteria

- Diagnosed with specific diseases, including peptic ulcer, esophageal cancer, MALT lymph cancer, stomach cancer, colon cancer, biliary tract and pancreatic diseases (except asymptomatic cholelithiasis), and inflammatory bowel disease within 1 year prior to the screening visit;
- Having more severe symptoms of reflux esophagitis and irritable bowel syndrome than those of dyspepsia (Participants with reflux esophagitis or irritable bowel syndrome can participate in this clinical trial. However, through the participants’ interview and medical history taking, participants whose symptoms of reflux esophagitis (including reflux and heartburn) and irritable bowel syndrome (including abdominal pain or discomfort related to the pattern of defecation) are more severe than those of dyspepsia are excluded.);
- Having alarming symptoms such as severe weight loss, bloody stool, dysphagia, swallowing pain, and persistent vomiting;
- Diagnosed with mental disorders, such as major depressive disorder, anxiety disorder, and panic disorder, or having a depression score of 11 or higher on the Hospital Anxiety and Depression Scale (HADS) at the screening visit;
- Having serious systemic diseases in heart, lung, liver, or kidney, or presence of other malignant diseases;
- Having a history of gastrointestinal surgery (except for appendectomy more than 6 months ago);
- Taking medications that can affect the gastrointestinal tract, including proton pump inhibitors, histamine receptor antagonists, antacids, prokinetics, antidepressants, gastric fundus relaxants, gastric mucosal protectants, nonsteroidal anti-inflammatory drugs, anticholinergics, or oral corticosteroids, or receiving *Helicobacter pylori* eradication treatment, within 2 weeks prior to the screening visit (taking low-dose aspirin (100 mg/day or less) for the purpose of preventing cardiovascular disease is allowed);
- Having received Korean medicine treatments such as acupuncture, herbal medicine, or moxibustion for the purpose of improving dyspeptic symptoms within 2 weeks prior to the screening visit, or who are planning to receive them during the trial period;
- Having severe liver or renal diseases (aspartate aminotransferase or alanine aminotransferase levels ≥ 3 times the upper limit of normal levels or creatinine levels ≥ 2 times the upper limit of normal levels);
- Having a history of alcohol or drug abuse;
- Women who are pregnant or lactating, or women who do not agree to use effective methods of contraception during the clinical trial;
- Having genetic problems, such as galactose intolerance, Lapp lactase deficiency, or glucose-galactose malabsorption;
- Having known hypersensitivity to investigational products (according to the Korean Ministry of Food and Drug Safety (KMFDS), adverse events such as pseudoaldosteronism, myopathy, and skin reactions (rash, redness, itching, hives) may occur after the administration of YGJ and PWS.);
- Having taken other investigational products within 3 months; and
- Having judged to be unsuitable for participation in this trial by investigators.

### 2.5. Dropout and Early Termination Criteria

At any time during the clinical trial, participants can voluntarily withdraw from the clinical trial. Additionally, anyone who meets any of the following conditions will be removed from the trial at the discretion of investigators.

- Found to violate the inclusion and exclusion criteria after screening visit;
- In the case where the participant requests discontinuation of the trial or refuses the treatment due to unsatisfactory treatment effects during the trial period;
- In the case where the participant cannot be tracked during the trial period;
- When it is judged by investigators that trial participation is no longer possible due to an adverse reaction or a concomitant disease;
- In the case of taking prohibited drugs that may affect the trial result without the instruction of investigators during the trial period;
- When it is judged that the effect evaluation of the clinical trial is not appropriate because the overall compliance with the investigational products is less than 70% in YGJ and PWS groups;
- When the participant violates the trial plan, and it is difficult for the participant to continue the trial; and
- When investigators judged that the progress of the clinical trial is no more appropriate.

In principle, this clinical trial will be finalized when the registration of the planned number of participants is completed and the completeness of the collected data is secured. However, this clinical trial can be discontinued in the following cases: (1) if the supplied investigational products are found to be harmful, (2) if moderate or severe adverse events judged to be related to the investigational products occur in more than 25% of all participants, and (3) in the case when the targeted number of participants is not registered even though a sufficient recruitment period has been given.

### 2.6. Randomization and Allocation Concealment

Participants will be randomly assigned to the YGJ, PWS, and usual care groups at a 2:2:1 ratio at the baseline visit (Visit 2). This allocation ratio was set in consideration of previous research [11,19] and ethical aspects. A random number will be generated using SAS^®^ Version 9.4 software (SAS Institute Inc., Cary, NC, USA) by a statistician (O.J.K.) independent of the trial procedure and assessment. The block randomization method will be used in block sizes of 5 and 10 with no stratification. For allocation concealment, the randomization code will be sealed in opaque envelopes and stored in locked cabinets in each trial institution. If the participants who voluntarily signed the written informed consent are able to participate in this clinical trial by meeting the eligibility criteria, the investigator opens the randomization envelope in front of the participants in order and assigns them to one of three groups. The date of opening and the signature of the investigator will be written on the opened envelope, which will be stored separately.

### 2.7. Blinding

Because it is not possible to blind the participants due to the nature of group assignment, we designed the trial to blind the outcome assessor to minimize the related bias. The effect evaluation of the participants will be conducted by an investigator who will not prescribe the investigational product and will not have access to any source documents, including medical records and case report form (CRF). The outcome assessor only asks questions related to the evaluation items and does not know which group the clinical trial participants are assigned to.

### 2.8. Interventions

Participants will be randomly assigned to receive YGJ, PWS, or usual care for 6 weeks. All investigational drugs in the YGJ and PWS groups were manufactured by Kracie Pharma Korea Co., Ltd. (Seoul, Republic of Korea) and Hanpoong Pharm & Foods Co., Ltd. (Jeonju, Republic of Korea), respectively, in compliance with the Korean Good Manufacturing Practice guidelines.

YGJ (Kracie Yukgunja-tang Extract Fine Granule; product code: 201310401) is a light-brown-to-brown-colored fine granule, and participants will consume one pack (3.0 g) twice a day before or between meals for 6 weeks. The raw materials of YGJ extract per pack are Ginseng Radix 2 g; Atractylodis Rhizoma Alba 2 g; Poria Sclerotium 2 g; Pinelliae Tuber 2 g; Citri Unshius Pericarpium 1 g; Zizyphi Fructus 1 g; Zingiberis Rhizoma Recens 0.25 g; and Glycyrrhizae Radix et Rhizoma 0.5 g. PWS (Hanpoong Pyeongwi-san, mixed single extract; product code: 200000079), an active control, is a brown-colored powder, and participants will consume one pack (2.18 g) three times a day before or between meals for 6 weeks. The component per 1 pack is a powder extract of Atractylodis Rhizoma 0.95 g; Citri Unshius Pericarpium 0.56 g; Magnoliae Cortex 0.10 g; Glycyrrhizae Radix et Rhizoma 0.20 g; Zingiberis Rhizoma Recens 0.03 g; and Zizyphi Fructus 0.34 g. All investigational products will be stored at room temperature, and a pharmacist will check their storage, dispensation, and quality. During the administration of the investigational products, investigators will check the number of remaining drugs to ensure that the participants have taken the drugs properly (Visits 3 and 4). Medication compliance (%) will be calculated by dividing the number of drugs actually taken by the number of drugs that should have been taken.

During the clinical trial period, dietary guidelines based on dietary recommendations prepared for Korean dyspepsia patients [20] will be distributed and explained to all participants in the three groups. The usual care group will perform daily management while following the dietary guidelines distributed during the clinical trial period without administration of any investigational product. Under the judgement of the investigator, medications and treatments other than those prohibited in this clinical trial may be permitted, and all medications and treatments performed during the trial period should be recorded in the CRF. YGJ and PWS groups are also allowed to perform usual care.

Concomitant medications are permitted only if the investigators determine that they do not affect the outcomes of this study, including drugs used to treat underlying diseases or therapeutic drugs in case of adverse events. The investigators will check and record all the detailed information of concomitant medications in the CRF. During the entire trial period, participants are prohibited from taking medications, including proton pump inhibitors, histamine receptor antagonists, antacids, prokinetics, antidepressants, gastric fundus relaxants, gastric mucosal protectants, nonsteroidal anti-inflammatory drugs, anticholinergics, oral corticosteroids, and treatment regimens for *Helicobacter pylori*, which, in the opinion of the investigators, may affect the gastrointestinal tract. In addition, it is prohibited to receive Korean medicine treatments such as acupuncture, herbal medicine, or moxibustion for the purpose of improving indigestion during the clinical trial period.

### 2.9. Outcome Measures

#### 2.9.1. Demographic and Baseline Information

Demographic information, including date of birth, age, sex, history of smoking, drinking, caffeine consumption, and exercise habits, will be investigated and recorded in the CRF. Socioeconomic characteristics such as educational background, conjugality, occupation, religion, and household income will also be collected for economic analysis. The morbidity period of FD and recent upper endoscopy findings will be investigated, and the subtypes of FD—epigastric pain syndrome (EPS), postprandial distress syndrome (PDS), and the overlap of the two subgroups—will be determined according to Rome IV. PDS is a condition in which there is a feeling of fullness after a meal or bothersome early satiety, whereas EPS is characterized by unpleasant epigastric pain or burning, not necessarily related to food intake. The clinically significant medical history and surgery history within the last 1 year and treatment history within the last 4 weeks of the participants will be investigated and recorded in the CRF.

In addition, deficiency and excess pattern identification will be evaluated and recorded under the judgment of a Korean medicine doctor. Investigators will perform a systemic physical examination; collect baseline characteristics, including height, weight, and body mass index; and check for infection of *Helicobacter pylori* using Anti-*Helicobacter pylori* IgG serology at the screening visit. Furthermore, the participants will assess their health status related to FD for the past two weeks using a standard tool for pattern identification of FD [21] Then, they will be classified into the following six pattern identifications: spleen–stomach deficiency and cold, spleen deficiency with qi stagnation, liver–stomach disharmony, cold–heat complex, spleen–stomach dampness and heat, and dietary retention and stagnation.

#### 2.9.2. Primary Outcome: Total Dyspepsia Symptom Scale (TDS)

The TDS questionnaire evaluates eight items consisting of postprandial fullness/bloating, early satiety, epigastric pain and burning, nausea, vomiting, belching, and other dyspeptic symptoms on a 4-point Likert scale (0: none, 1: mild/weak, 2: moderate/average, and 3: severe) [11]. A higher overall score, obtained by adding up the points for each item, indicates more severe symptoms related to indigestion. The TDS has been used as a primary outcome in a number of clinical trials related to FD [11,19,22]. Therefore, the TDS will be measured at all visits except the screening visit. The degree of improvement in the TDS score during the trial period will be calculated using the following formula:

$$\mathrm{Improvement}\;\mathrm{in}\;\mathrm{TDS}\;\left(\%\right)=\frac{TDS\;at\;baseline-TDS\;at\;N\;th\;week}{TDS\;at\;baseline}\times 100$$

The therapeutic effects will be classified as significant (≥75%), moderate (≥50%), mild (≥25%), and none (<25%) according to the improvement rate.

#### 2.9.3. Secondary Outcomes

(1) Single Dyspepsia Symptom Scale (SDS)

The SDS questionnaire evaluates the frequency, intensity, and bothersomeness of the most common symptoms of FD, using a 4-point Likert scale, i.e., by measuring the following symptoms: postprandial fullness/bloating, early satiety, and epigastric pain and burning [11]. A higher sum of scores corresponding to the symptoms means more severe indigestion. SDS will be obtained at all visits except the screening visit and telephone follow-up (Visit 6). The degree of improvement in the SDS score will be calculated in the same way as that of the TDS.

(2) Overall Treatment Effect (OTE)

Using the OTE questionnaire, the subjectively perceived therapeutic effect on dyspeptic symptoms and quality of life will be assessed. At all visits after allocation, participants will be asked about how much their symptoms improved compared to before participating in the clinical trial, by answering with one of the following 7 items: “very much worse,” “much worse,” “slightly worse,” “no change,” “slightly improved,” “much improved,” and “very much improved” [12]. The response rate for each item will be calculated, and participants who responded “much improved” or “very much improved” will be defined as responders.

(3) VAS for Dyspepsia

Participants will check the intensity of dyspepsia symptoms over the past 2 weeks themselves using a VAS in a horizontal line drawn from 0 (not uncomfortable at all) to 100 (the worst dyspepsia you can imagine). The scores will be obtained at all visits except baseline (Visit 2) and telephone follow-up (Visit 6).

(4) FD-related Quality of Life (FD-QoL)

The FD-QoL is a questionnaire used to evaluate the quality of life of patients with FD, which consists of 21 items in four dimensions, including psychological, role-functioning, eating, and liveliness status. It has been validated for Korean patients with FD, where lower scores represent a worse quality of life [23]. Data will be collected at baseline (Visit 2), week 3 (Visit 3), and week 6 (Visit 4).

(5) HADS

The HADS questionnaire has a total of 14 items and consists of seven anxiety subscales and seven depression subscales. Each item is evaluated on a 4-point scale, and a higher score means more complaints of symptoms [24]. It will be measured at all visits except baseline (Visit 2) and telephone follow-up (Visit 6).

(6) Five-level Version of the EuroQol Five-dimensional Descriptive System (EQ-5D-5L)

The EQ-5D-5L is a self-reporting questionnaire applied to evaluate quality of life. It comprises five dimensions as follows: mobility, self-care, usual activities, pain/discomfort, and anxiety/depression [25]. In addition, participants’ self-rated health status will be recorded on a vertical VAS scale drawn from 0 (the worst health you can imagine) to 100 (the best health you can imagine). It will be assessed at all visits except the screening visit.

(7) Spleen Qi Deficiency Questionnaire (SQDQ)

The SQDQ consists of 11 items associated with symptoms such as loss of appetite, reduced food intake, and general fatigue, evaluated on a 5-point scale [26]. After applying weight conversion to each question, if the total score exceeds 43.18 points, the participants will be diagnosed as spleen qi deficiency [27]. Data will be collected at baseline (Visit 2), week 3 (Visit 3), and week 6 (Visit 4).

(8) Damum Questionnaire (DQ)

The DQ is an instrument for determining if a participant has symptoms of phlegm pattern such as dizziness, chest discomfort, and indigestion. It includes a total of 14 items on the basis of a 7-point scale. Higher scores calculated by multiplying the weights indicate a high possibility of the existence of Damum [28]. The DQ will be evaluated at baseline (Visit 2), week 3 (Visit 3), and week 6 (Visit 4).

(9) Serum Ghrelin Levels

We will evaluate serum levels of acyl-ghrelin and deacyl-ghrelin, and the ratio of acyl-ghrelin to total ghrelin by collecting serum acyl-ghrelin and deacyl-ghrelin at the screening visit and week 6 (Visit 4).

#### 2.9.4. Safety Outcomes

The following laboratory parameters will be measured to assess the safety of investigational products at the screening visit and week 6 (Visit 4) in all participants: liver function test (aspartate aminotransferase and alanine aminotransferase), renal function test (blood urea nitrogen and creatinine), complete blood count, and electrolyte content (sodium and potassium). In addition, adverse events will be investigated through participants’ self-reporting and medical examination during the trial period, and the investigator will measure the vital signs (blood pressure, pulse rate, and body temperature) of participants at each visit.

### 2.10. Sample Size Calculation

The primary objective of this study is to compare and evaluate the effectiveness of 6 weeks of administration of YGJ compared to usual care in participants with FD. We will test whether there is a difference in the degree of reduction in TDS scores between the two groups after 6 weeks through a superiority test. Based on the results of a previous study comparing YGJ and a placebo [19], and considering the 15% placebo effect, it is assumed that the difference between the average TDS change between the YGJ and usual care groups after 6 weeks is 2.70, and the standard deviation is 3.65. The significance level is 0.05, and the power is 80%. Taking into account the previous studies [11,19] and ethical issues, the ratio between the YGJ and usual care groups is assigned at 2:1. Therefore, 44 and 22 participants are required for the YGJ and usual care groups, respectively.

Additionally, the second purpose of this study is to evaluate the noninferiority of YGJ compared with that of PWS. For this, the noninferiority margin should be defined based on the results of the effect evaluation between the PWS and usual care groups. However, due to the lack of prior research and related data, we will first test the superiority of PWS compared to usual care after 6 weeks to define the noninferiority margin. Under the assumption that the effect size of the YGJ and PWS is the same, 44 participants are also required in the PWS group. Therefore, considering the 20% dropout rate, a total of 140 participants, including 56, 56, and 28 participants in the YGJ, PWS, and usual care groups, respectively, are required.

### 2.11. Statistical Analysis

A statistician independent of the trial procedure and assessment will conduct statistical analysis using SAS^®^ Version 9.4 software (SAS Institute Inc., Cary, NC, USA). In principle, for effectiveness analysis, a full analysis set of all randomly assigned participants who are evaluated at least once after receiving a prescription of investigational products at least once will be conducted, according to intention-to-treat analysis. If necessary, per-protocol set (PPS) analysis will be performed, including participants who have completed the entire process of the clinical trial and have no significant violations affecting the test results. Participants with less than 70% compliance with the investigational product, taking prohibited drugs for the treatment and management of diseases other than gastrointestinal disease under the judgment of investigators, or experiencing events that can cause digestive symptoms such as sudden stress during the clinical trial period, will be excluded from the PPS analysis. Safety analysis will include all data obtained from participants who have taken at least one investigational product in the YGJ and PWS groups and all participants in the usual care group.

The demographic characteristics of participants will be summarized by group. Continuous data will be presented as mean and 95% confidence intervals (CIs), whereas dichotomous data will be displayed as frequency and percentage. The primary outcome of this study is the difference in the TDS score at week 6 between the YGJ and usual care groups (Hypothesis 1).

**Hypothesis** **1.** *[* $H_{0}\;:\;\mu_{1}=\mu_{2},\;H_{1}\;:\;\mu_{1}\neq \mu_{2}$ *]*

*-* $\mu_{1}$ *: Change in TDS score of the YGJ group at week 6*

*-* $\mu_{2}$ *: Change in TDS score of the usual care group at week 6*

A two-sided test with a significance level of 5% will be conducted using a mixed-effect model repeated measure (MMRM) with participants as the random factor and the treatment group and time of visit as the fixed factors. If necessary, analyses will be performed by setting variables showing statistically significant differences in demographic characteristics or variables that can affect dyspepsia as the fixed factors. The second purpose of this study is to evaluate the noninferiority of YGJ compared to that of PWS (Hypothesis 3). Consequently, the noninferiority margin (*M*) will first be defined based on the results of the effect evaluation of PWS compared to usual care at week 6 (Hypothesis 2), and the analysis method will be the same as that of Hypothesis 1.

**Hypothesis** **2.** *[* $H_{0}\;:\;\mu_{3}=\mu_{2},\;H_{1}\;:\;\mu_{3}\neq \mu_{2}$ *]*

*-* $\mu_{2}$ *: Change in TDS score of the usual care group at week 6*

*-* $\mu_{3}$ *: Change in TDS score of the PWS group at week 6*

**Hypothesis** **3.** *[* $H_{0}\;:\;\mu_{3}-\mu_{1}>M,\;H_{1}\;:\;\mu_{3}-\mu_{1}\;\leq M$ *]*

*-* $\mu_{1}$ *: Change in TDS score of the YGJ group at week 6*

*-* $\mu_{3}$ *: Change in TDS score of the PWS group at week 6*

To evaluate the noninferiority of YGJ compared to PWS, Hypothesis 3 will be tested in the same way as Hypothesis 1. However, since the noninferiority test is a single test, the significance level will be set to 2.5%, and the noninferiority margin will be defined as the lower limit of the 95% CI of Hypothesis 2. The multiple test problem will be solved using the fixed sequence method [29]. In other words, if the statistical test result for Hypothesis 1 is significant, the test for Hypothesis 2 will be performed, and if the statistical test result for Hypothesis 1 is not significant, the test for Hypothesis 2 and Hypothesis 3 will not be performed. If the statistical test result for Hypothesis 1 is significant and the test for Hypothesis 2 is performed, and if the statistical test result for Hypothesis 2 is significant, the test for Hypothesis 3 will be performed. If the statistical test result is not significant, the test for Hypothesis 3 will not be performed. If possible, subgroup analysis will be performed by categorizing the participants’ baseline characteristics, including age (65 years old or older/under), Rome IV subtype (EPS, PDS, and overlap), type of FD pattern identification (according to the standard tool for pattern identification of FD [21]: spleen–stomach deficiency and cold, spleen deficiency with qi stagnation, liver–stomach disharmony, cold–heat complex, spleen–stomach dampness and heat, and dietary retention and stagnation), deficiency and excess pattern identification, and the presence or absence of *Helicobacter pylori* infection and spleen qi deficiency syndrome (cut off score: 43.18 points in SQDQ [27]). In addition, we will combine the results of the YGJ and PWS groups corresponding to herbal medicine and compare them with the usual care control group.

Other secondary outcome measures will be tested in the same way as the primary outcome measure. In addition, to compare the difference between the values before and after treatment in each group, the continuous variable will be analyzed using Student’s paired *t*-test or Wilcoxon signed-rank test, and the discrete variable will be Fisher’s exact test. Repeated measures analysis of variance will be performed to compare the differences in trend change in each group of visits, and Dunnett’s procedure (based on the baseline) will be used as a multiple comparison correction.

### 2.12. Economic Evaluation

We will conduct an economic evaluation along with the RCT for effectiveness and safety. We will estimate the cost-effectiveness of YGJ compared with PWS or usual care from the health care system and societal perspective. Costs will consist of medical costs, nonmedical costs (transportation costs, time costs, and care costs), and costs associated with productivity loss using the Work Productivity and Activity Impairment Questionnaire: General Health V2.0, which is the most frequently used instrument for measuring productivity loss in Korea [30,31]. Cost data will be obtained from each participant using a separately developed cost questionnaire and institutional data at all visits except at the screening visit. However, if necessary, data from Statistics Korea can be used. Effectiveness data will be collected from the main RCT. Utility data will be collected from the main RCT using the EQ-5D-5L questionnaire, and quality-adjusted life years gained will be calculated using the area under the curve method [25]. We will investigate the cost and effectiveness data of TDS, OTE, and EQ-5D-5L for economic evaluation through telephone follow-up at week 24, even after the end of the 9-week clinical trial for effectiveness and safety evaluation of YGJ. These effectiveness, utility, and cost data will be used to calculate the incremental cost-effectiveness (cost–utility) ratio between the YGJ and the control groups at each visit. If necessary, we will perform a one-way sensitivity analysis on possible variables and present a tornado diagram, and also perform a probability sensitivity analysis using the distribution and representative values of the variables. In addition, subgroup analysis will be conducted according to the RCT subgroup analysis plan of the effectiveness outcome measures. Among the dropout criteria of the clinical trial for effectiveness evaluation, if the investigator judges that it is no longer possible for a participant to participate in the trial due to an adverse reaction or comorbid disease in the clinical trial process, participants will not be excluded from the data collection of the economic evaluation, and a telephone follow-up survey will be performed at week 24 with the consent of the participants. If we cannot observe the full yield cost and effectiveness outcomes of interventions during the 6 months clinical trial period, we will develop a decision-analytic model and conduct a long-term evaluation by secondary analysis.

### 2.13. Data Management and Monitoring

A web-based CRF iCReaT (Osong, Republic of Korea) will be used for data collection and verification, and will be managed by an independent data management team from the Korea Institute of Oriental Medicine (KIOM). It is only accessible to investigators who are directly involved in this clinical trial and have received the relevant education. Although data entry will be performed only once by the investigators in each trial institution, data quality will be managed using two verification processes for each clinical research associate (CRA) and data manager (DM) of KIOM.

For data monitoring, CRAs from KIOM, a supporting institution, will visit the trial institutions regularly to confirm and check compliance with the clinical trial protocol, appropriate and accurate data collection, participants’ informed consent, adverse events, and management of investigational products during the trial period. No interim analysis has been planned. The trial will be terminated after all the planned participants have been recruited, relevant data have been collected, and CRA and DM have checked and verified all the data. There are currently no planned audits.

### 2.14. Patient and Public Involvement

YGJ and PWS are herbal drugs approved by KMFDS in the Republic of Korea and have been used for the treatment of FD for many years. Hence, these medicines are easily accepted by patients. In particular, PWS is an herbal drug covered by health insurance benefits for patients with dyspepsia. We will use the patient’s subjective dyspeptic symptom intensity as the primary endpoint of our study, which is an important criterion in the clinical setting. However, patients were not involved directly in developing the trial protocol. Although there is no plan to communicate the study results to participants, we will provide a summary to the participants upon request after the completion of the clinical trial. Additionally, participants can contact investigators at any time to obtain information related to the clinical trial.

### 2.15. Ethics and Dissemination

The trial protocol has been approved by the IRBs of Kyung Hee University Korean Medicine Hospital (KOMCIRB 2021-01-001), Daejeon Korean Medicine Hospital of Daejeon University (DJDSKH-21-DR-03), and Semyung University Korean Medicine Hospital (SMJOH-2021-01). The protocol of this trial was registered at the Clinical Research Information Service (CRIS) (registration number: KCT0006044) before the enrollment of the first participant. Any modifications to the protocol will be reapproved by the IRBs and documented in CRIS. A detailed description of the trial process will be provided to participants by licensed Korean medicine doctors and, participants will be asked to sign an informed consent form prior to participation in the trial. The results of this study will be published in peer-reviewed journals and can be disseminated through conference presentations.

## 3. Discussion

The present study is a protocol for a randomized, assessor-blind, multicenter, controlled clinical trial evaluating the effectiveness and safety of YGJ compared with usual care and PWS in patients with FD, and will be conducted in conjunction with a cost-effectiveness analysis. We aim to test the superiority of YGJ to usual care and noninferiority to PWS for the treatment of FD. In addition, although YGJ is actively used in the Korean medicine clinical field for the treatment of FD, a cost-effectiveness analysis has not yet been conducted for this. We intend to prepare the basic data for health insurance benefit registration of YGJ by confirming the cost-effectiveness of YGJ compared to PWS, the most commonly used health insurance benefiting herbal medicine for the treatment of FD in Korean medicine clinical settings [7]. Based on consultation with Korean medicine gastroenterology experts and prior literature that stated that at least 4–6 weeks of treatment was required to see the significant therapeutic effect of YGJ, we set 6 weeks as the administration period of investigational products [12].

According to the guideline recommendations for outcome measures used in clinical trials for FD [32], the primary efficacy outcome measures to be used in FD clinical trials are diverse and have not yet been standardized. Additionally, in Korea, a patient-reported outcome measure that has been verified as recommended by local or global guidance has not yet been developed. Accordingly, it is recommended to use the total symptom score and OTE as the primary efficacy endpoint for FD clinical trials [32]. Therefore, in our study, the TDS score, which was used frequently as the primary outcome measure in previous studies on FD [11,19,22], was selected as the primary outcome according to the expert consensus, and OTE was set as the secondary outcome. Additionally, based on such previous studies [11,19], a sample size suitable for hypothesis testing was calculated in the present study.

In addition, based on previous studies showing that psychological factors are closely related to the pathogenesis of FD [12,33,34], we will measure HADS as a secondary outcome to determine whether anxiety and depressive symptoms are improved after administration of investigational products and to examine the correlation with other outcomes such as TDS and ghrelin.

According to the traditional Korean medicine theory, YGJ is a kind of herbal medicine that treats spleen qi deficiency and dampness-phlegm. We will also evaluate SQDQ and DQ as outcome measures to see if the symptoms of spleen qi deficiency and dampness-phlegm improve after 6 weeks of YGJ administration.

YGJ is a representative herbal medicine widely used in patients with FD. In recent years, experimental and clinical studies have been actively conducted to study the pharmacological actions of YGJ. In particular, various studies have been published showing that YGJ improves FD by enhancing ghrelin. However, due to the small number of specimens, no definite conclusion can be drawn [13,35]. Additionally, to the best of our knowledge, no studies have been conducted on the effect of PWS on ghrelin levels in improving FD symptoms. In addition to the self-reported subjective outcomes, we will measure changes in acyl-ghrelin, deacyl-ghrelin, and total ghrelin (acyl-ghrelin plus deacyl-ghrelin) levels before and after administration of the investigational products, which will also be used to explore different mechanisms of action for improving dyspepsia due to YGJ and PWS.

The YGJ and PWS which will be used in this clinical trial are approved by KMFDS. According to a previous study in which participants with FD were administered YGJ for 8 weeks [13], diarrhea (seven cases), nausea (three cases), headache (three cases), increase in the level of gamma-glutamyl transpeptidase (two cases), epigastric pain (two cases), increase in the level of alanine aminotransferase (one case), abdominal bloating (one case), abdominal discomfort (one case), common cold (one case), tinnitus (one case), skin abnormal sensation (one case), oral abnormal sensation (one case), vertigo (one case), and urticaria (one case) were reported as adverse events. There was no significant difference in the incidence of adverse reactions between the YGJ and the placebo groups, and no serious adverse reactions were reported in either group. In addition, in the previous study [17] in which modified PWS was administered for 8 weeks in participants with FD, no adverse reactions related to the PWS administration occurred. Therefore, we judged that the administration of YGJ or PWS for 6 weeks in this clinical trial is unlikely to cause serious harm to the participants.

One limitation of this study is that it is restricted to the Korean population, which may make the generalization of the results to other populations around the world difficult. However, the results of this study will prove the effectiveness and cost-effectiveness of YGJ, a non-covered herbal medicine for FD, in accordance with the national health insurance situation in Korea, thereby helping to expand the medical options and alleviate the health burden of Korean FD patients. The results of our study will provide strong evidence of YGJ, an important option for the treatment of FD, to clinicians, patients, researchers, and policy-makers.

## Figures and Tables

**Table 1:** Schedule of enrolment, interventions, and assessments.

	Study Period					
	Enrolment	Allocation	Medication		Follow-Up	
Procedures	Visit 1 (Screening)	Visit 2	Visit 3	Visit 4	Visit 5	Visit 6 (Telephone)
TIMEPOINT	−2 weeks	Week 0	Week 3 ±4 days	Week 6 ±4 days	Week 9 ±4 days	Week 24 ±4 days
ENROLLMENT						
Eligibility screen	●					
Informed consent	●					
Demographics	●					
Medical and treatment history	●					
Physical examination	●					
Vital signs	●	●	●	●	●	
PIFD	●					
Allocation		●				
INTERVENTIONS						
YGJ or PWS		○	○	○		
Dietary guideline education		●	●	●		
ASSESSMENTS						
TDS		●	●	●	●	●
SDS		●	●	●	●	
OTE			●	●	●	●
VAS (dyspepsia)	●		●	●	●	
FD-QoL		●	●	●		
HADS	●		●	●	●	
EQ-5D-5L EQ-VAS		●	●	●	●	●
SQDQ		●	●	●		
DQ		●	●	●		
Cost questionnaire		●	●	●	●	●
Laboratory tests *	●			●		
Adverse events investigation		●	●	●	●	
Compliance test			○	○		

●: all participants, ○: YGJ and PWS groups, * Including hematological, blood glucose level, liver and renal function tests, electrolyte test, acyl-ghrelin, and deacyl-ghrelin. In addition, human chorionic gonadotropin urine test for women in their childbearing years, and anti-*Helicobacter pylori* serum IgG testing at the screening visit. Abbreviations. DQ, Damum questionnaire; EQ-VAS, the EuroQol visual analog scale; EQ-5D-5L, five-level version of the EuroQol five-dimensional descriptive system; FD-QoL, functional dyspepsia-related quality of life; HADS, hospital anxiety and depression scale; OTE, overall treatment effect; PIFD, pattern identification of functional dyspepsia; PWS, Pyeongwi-san; SDS, single dyspepsia symptom; SQDQ, spleen qi deficiency questionnaire; TDS, total dyspepsia symptom; VAS, visual analog scale; YGJ, Yukgunja-tang.

## Data Availability

No datasets were generated or analyzed during the current study. All relevant data from this study will be made available upon study completion.
